# Prognostic Value of the Interaction between Galectin-3 and Antigen Carbohydrate 125 in Acute Heart Failure

**DOI:** 10.1371/journal.pone.0122360

**Published:** 2015-04-13

**Authors:** Julio Núñez, Gabriel A. Rabinovich, Justo Sandino, Luis Mainar, Patricia Palau, Enrique Santas, Maria Pilar Villanueva, Eduardo Núñez, Vicent Bodí, Francisco J. Chorro, Gema Miñana, Juan Sanchis

**Affiliations:** 1 Servicio de Cardiología, Hospital Clínico Universitario de Valencia, Universitat de Valencia, Valencia, Spain; 2 Laboratorio de Inmunopatología, Instituto de Biología y Medicina Experimental (IBYME), Consejo Nacional de Investigaciones Científicas y Técnicas (CONICET) and Facultad de Ciencias Exactas y Naturales (FCEyN), Universidad de Buenos Aires, Buenos Aires, Argentina; 3 Servicio de Cardiología, Hospital de la Plana, Villa-real, Spain; 4 Servicio de Bioquímica Clínica, Hospital Clínico Universitario de Valencia, Valencia, Spain; 5 Servicio de Cardiología, Hospital de Manises, Manises, Spain; Fondazione G. Monasterio, ITALY

## Abstract

**Aims:**

Galectin-3 (Gal-3) and carbohydrate antigen 125 (CA125) have emerged as robust prognostic biomarkers in heart failure. Experimental data have also suggested a potential molecular interaction between CA125 and Gal-3; however, the biological and clinical relevance of this interaction is still uncertain. We sought to evaluate, in patients admitted for acute heart failure, the association between plasma Gal-3 with all-cause mortality and the risk for rehospitalizations among high and low levels of CA125.

**Methods and Results:**

We included 264 consecutive patients admitted for acute heart failure to the Cardiology Department in a third-level center. Both biomarkers were measured on admission. Negative binomial and Cox regression models were used to evaluate the prognostic effect of the interaction between Gal-3 and CA125 (dichotomized by its median) with hospital readmission and all-cause mortality, respectively. During a median follow-up of 2 years (IQR = 1-2.8), 108 (40.9%) patients deaths and 365 rehospitalizations in 171 (69.5%) patients were registered. In a multivariable setting, the effect of Gal-3 on mortality and rehospitalization was differentially mediated by CA125 (p = 0.007 and p<0.001, respectively). Indeed, in patients with CA125 above median (>67 U/ml), values across the continuum of Gal-3 showed a positive and almost linear relationship with either the risk of death or rehospitalization. Conversely, when CA125 was below median (≤67 U/ml), Gal-3 lacked any prognostic effect on both endpoints.

**Conclusion:**

In patients with acute heart failure, Gal-3 was strongly associated with higher risk of long-term mortality and repeated rehospitalizations, but only in those patients exhibiting higher values of CA125 (above 67 U/ml).

## Introduction

Plasma galectin-3 (Gal-3) and carbohydrate antigen 125 (CA125) are two biomarkers up-regulated in heart failure (HF) [1–5]. Even though their biological roles are incompletely understood, both biomarkers have been shown to be associated with the severity and prognosis of the disease [1–5]. Indeed, Gal-3, an endogenous pro-inflammatory lectin, has been suggested to play a crucial role as a mediator of HF remodeling [1–3, 6], while CA125 has been identified as a potential surrogate for fluid overload and heightened inflammatory status [3, 7]. CA125 is a large glycoprotein synthesized by epithelial cells in response to diverse stimuli. Specifically, in HF, increased values of CA125 are frequently observed during acute decompensations [3, 4].

Gal-3 is a ubiquitous glycan-binding protein composed of approximately 30–35 kDa that contains a carbohydrate-recognition domain (CRD) enabling specific binding to glycosylated molecules [8]. Through specific interactions with glycosylated proteins, Gal-3 can mediate the formation of supramolecular structures on cell surfaces termed ‘lattices’, strengthening the avidity and half-life of ligand-receptor interactions, and organizing specialized clusters for molecular signaling [9]. Recent evidence identified CA125 as a specific binding partner of soluble lectins including galectin-1 (Gal-1) and Gal-3 [10]. This functional interaction has demonstrated to depend on β-galactose-terminated, *O*-linked oligosaccharide chains of CA125, and was found to be regulated by the cellular milieu in which CA125 is particularly expressed [10]. Not surprisingly, both Gal-3 and CA125 have been associated with similar pathophysiologic processes including regulation of cell adhesion, apoptosis, cell proliferation, inflammation, and tumour progression among others [1, 3, 7, 11, 12]. Nevertheless, the cell signalling events and pathophysiologic processes arising from Gal-3-CA125 interactions have not been well explored; although they seem to play a key biological role in maintaining mucosal barrier function, and facilitating malignant progression [8–12]. As both biomarkers are mechanistically interrelated, we speculated that the deleterious effect of Gal-3 in HF might be influenced by the relative plasma levels of CA125. In this work we aimed to evaluate, in an unselected cohort of patients admitted for acute heart failure (AHF), the independent association between Gal-3 with long-term mortality and with repeated readmissions according to their plasma concentration of CA125.

## Methods

This study was approved by the institutional review committee: Comité Ético de Investigación Clínica (CEIC) Hospital Clínico Universitario de Valencia. All patients provided their written consent to participate in this study

### Study sample

We included 264 consecutive patients admitted to the Cardiology Department of a third level center (Hospital Clínico Universitario de Valencia, Spain) from November 11th 2010 to July 1st 2012. AHF diagnosis was made by trained cardiologists, and according to the definition proposed by guidelines of the European Society of Cardiology [13]. Either patients with new onset AHF or decompensated chronic HF were eligible. By design, patients with a discharge diagnosis of acute coronary syndrome, pneumonia, cancer or pulmonary thromboembolism were excluded. Upon patient admission, information related to demography, medical history, vital signs, 12-leads electrocardiogram, lab data, and pharmacologic therapies were routinely recorded following pre-established registry questionnaires. Lab data included blood chemistry, renal function parameters, amino-terminal pro-brain natriuretic peptide (NT-proBNP), high-sensitivity troponin T (Hs-TnT), high-sensitivity C-reactive protein (Hs-CRP), plasma levels of interleukin (IL)-1β (IL-1β), IL-6, tumor necrosis factor-α (TNF-α) and IL-10 were also measured on admission. Left ventricular ejection fraction (LVEF) and routine laboratory tests were also assessed during hospitalization. All patients were treated with intravenous furosemide at least during the first 48 hours of admission. Additional medical treatment with nitrates, inotropes, angiotensin converting enzyme inhibitors (ACEI), angiotensin receptor blockers (ARB), beta-blockers, aldosterone antagonists, anticoagulants and other therapeutic strategies were individualized following established guidelines and/or physician preferences [13]. This study was approved by the institutional review committee and all patients gave informed consent.

### Measurements of plasma Gal-3, CA125 and cytokines

Plasma Gal-3, CA125, IL-1β, IL-6, IL-10 and TNF-α were obtained in the first blood sample obtained at emergency room arrival and were subsequently processed using commercially available assays [VIDAS Galectin-3-Biomérieux, HSCYTMAG-60SK (High Sensitivity Human Cytokine Magnetic panel Milliplex and Elecsys CA125 II assay-Roche Diagnostics for Gal-3, CA125 and cytokines, respectively].

### Follow-up and endpoints

Patients’ follow-up was censored if death or valve replacement ensued. Long-term all-cause mortality and repeated unplanned rehospitalizations were selected as the main endpoints. Secondary endpoints were mortality and readmission due to cardiovascular (CV) causes. Clinical endpoints were verified through electronic patients’ clinical chart, and adjudicated by an investigator who was blinded to patient’s levels of both biomarkers.

### Statistical analysis

Continuous variables were expressed as mean ± 1 standard deviation (SD) or median (interquartile range) when appropriate. Discrete variables were summarized as percentages. Gal-3 was dichotomized based on a proposed prognostic cutpoint (17.8 ng/ml) [1, 2, 14]. CA125 was dichotomized by its median (67 U/ml), a value also reported as a prognostic cut point [3]. A variable with 4 categories was formed by combining these two variables: C1 = Gal-3 ≤17.8 ng/ml and CA125 ≤67 U/ml (n = 32); C2 = Gal-3 ≤17.8 ng/ml and CA125 >67 U/ml (n = 43); C3 = Gal-3 >17.8 ng/ml and CA125 ≤67 U/ml (n = 101); and C4 = Gal-3 >17.8 ng/ml and CA125 >67 U/ml (n = 88). Mortality rates among these categories were depicted with Kaplan-Meier method. Additionally, Gal-3 was dichotomized by its median value and a variable of 4 categories combining CA125 and Gal-3 were also evaluated.

According to the working hypothesis, the association between Gal-3 and all-cause mortality was differentially evaluated among two groups of patients: those with low or high levels of CA125 (≤67 U/ml and >67 U/ml, respectively). Such analysis was carried out by a Cox proportional hazard regression, and the results were expressed as hazard ratios (HR) with 95% confidence intervals (CI).

For the rehospitalization endpoint, we evaluated the effect of the same interaction (CA125 median and Gal-3) on the count of the number of rehospitalization that each patient had at follow-up. Due to the count nature of the endpoint, a negative binomial regression (NBreg) was used. To account for differences in follow-up, the log of the number of post-discharge years was included as model’s offset. Death, when occurred outside any rehospitalization, was added (as an additional event) to the number of readmissions to minimize the bias induced by mortality as a terminal endpoint (informative censoring) [15]. Robust standard errors were estimated to account for overdispersion and model’s misspecification.

For any regression model, candidate covariates were chosen based on previous medical knowledge. Then, a backward stepwise selection with AIC as stopping criterion was used to achieve a parsimonious model and thus prevent model overfitting. During this process, the linearity assumption for continuous variables was simultaneously tested, and transformed if appropriate, with fractional polynomials. The discriminative ability and the proportionality assumption for the hazard function over time were assessed by the C-statistics and the Schoenfeld residuals, respectively. For the NBreg model, the Explained Variance R^2^ and AIC were used as model’s fit criteria.

The final multivariable model for all-cause mortality included age, prior admission for AHF, prior history of stroke, wide QRS (>120 msec), systolic blood pressure, LVEF, estimated glomerular filtration rate, Hs-TnT, NT-proBNP and the interaction CA125-median*Gal-3. The Harrell´s C-statistics and the Groennesby and Borgan test for calibration were 0.771 and 0.359, respectively. Covariates included in the final multivariable model for the number of readmissions were Charlson comorbidity index, wide QRS (>120 msec), hemoglobin and the interaction CA125-median*Gal-3. The Explained Variance R^2^ of the NBreg model was 66.05%.

A 2-sided p-value of <0.05 was considered to be statistically significant for all analyses. All survival analyses were performed using STATA 13.1 (StataCorp. 2013. Stata Statistical Software: Release 13.1. College Station, TX: StataCorp LP).

## Results

The mean age was 72.7±11.3 years; 50.4% were female, 49.6% had prior history of HF, 36.4% were previously admitted for AHF and 55.3% exhibited LVEF ≥50%. The median (IQR) values for Gal-3, CA125 and NT-proBNP were 22.3 ng/ml (17.3–32), 67 U/ml (29–137) and 4813 pg/ml (2218–8618) respectively.

### Baseline profiles across Gal-3 and CA125 categories

Overall, patients with higher Gal-3 had a worse baseline risk profile. They were older, had longer length of stay, and had higher prevalence of comorbidity, renal dysfunction and elevated mean NT-proBNP. Likewise, they showed higher proportion of prior admission for AHF, signs of fluid overload and lower LVEF (Table 1). Among patients with elevated Gal-3 (>17.8 U/ml), those with higher values of CA125 had higher prevalence of fluid overload, valvular HF etiology, higher NT-proBNP and potassium, and a trend to lower tricuspid annular plane systolic excursion and sodium (Table 1).

**Table 1:** Baseline characteristics according Galectin-3/CA125 categories.

Variables	Galectin-3/CA125 categories				Omnibus p-value
	Low galectin-3		High galectin-3		
	Low CA125 (C1) (n = 32)	High CA125 (C2) (n = 43)	Low CA125 (C3) (n = 101)	High CA125 (C4) (n = 88)	
**Demographics and medical history**					
**Age, years**	72 ± 10	68 ± 12	75 ± 9	74 ± 10	0.004
**Male, n (%)**	16 (50)	19 (44.2)	49 (48.5)	50 (56.8)	0.523
**First admission for AHF, n (%)**	18 (56.2)	35 (81.4)	61 (60.4)	64 (61.4)	0.048
**LOS, days** ^**a**^	7.5 (4–9)	6 (4–9)	8 (6–13)	8 (6–13)	0.005
**Hypertension, n (%)**	28 (87.5)	27 (62.8)	83 (82.2)	76 (86.4)	0.014
**Diabetes Mellitus, n (%)**	16 (50)	21 (48.8)	48 (47.5)	46 (52.3)	0.932
**Dyslipidemia, n (%)**	18 (56.2)	22 (51.2)	61 (60.4)	50 (56.8)	0.783
**Current smoker, n (%)**	1 (3.13)	5 (11.6)	8 (7.9)	8 (9.1)	0.552
**History of alcohol abuse, n (%)**	2 (6.2)	4(9.3)	5(4.9)	4 (4.5)	0.743
**Ischemic heart disease, n (%)**	13 (40.3)	12 (27.9)	43 (42.6)	25 (28.4)	0.131
**Valvular heart disease, n (%)**	11 (34.4)	15 (34.9)	22 (21.8)	32 (36.4)	0.120
**Charlson index** ^**a**^	2 (1–3)	1 (0–2)	2 (1–4)	2.5 (1–4)	0.002
**COPD, n (%)**	8 (25)	4 (9.3)	26 (25.7)	25 (28.4)	0.063
**PAD, n (%)**	3 (9.4)	4 (9.3)	13 (12.9)	7 (7.9)	0.725
**Stroke, n (%)**	2 (6.2)	2 (4.5)	12 (11.9)	11 (12.5)	0.366
**Prior of history of heart failure, n (%)**	15 (46.9)	11 (25.6)	54 (53.5)	51 (57.9)	0.004
**Peripheral edema, n (%)**	20 (62.5)	32 (74.4)	62 (61.4)	75 (85.2)	0.001
**Pleural effusion, n (%)**	11 (34.4)	30 (69.8)	49 (48.5)	69 (78.4)	<0.001
**ICD, n (%)**	2 (6.2)	0	2 (2.0)	4 (4.5)	0.208
**Vital signs**					
**Heart rate, bpm**	100 ± 30	100 ± 31	96 ± 27	95 ± 27	0.717
**SBP, mmHg**	153 ± 41	142 ± 29	149 ± 32	148 ± 36	0.591
**DBP, mmHg**	86 ± 22	81 ± 18	81 ± 18	80 ± 18	0.466
**Electrocardiogram**					
**QRS >120 msec, n (%)**	14 (43.7)	10 (23.3)	43 (42.6)	36 (40.9)	0.123
**Atrial fibrillation, n (%)**	15 (46.9)	19 (44.2)	40 (39.6)	47 (53.4)	0.298
**Laboratory**					
**Hemoglobin, g/dl**	11.7 ± 2.0	12.4 ± 1.9	12.1 ± 1.9	12.1 ± 2.1	0.580
**WHO criteria for anemia, n (%)**	20 (62.5)	19 (44.2)	56 (55.4)	50 (56.8)	0.410
**Leucocyte count, 10** ^**3**^ **cells/ml**	9212 ± 351	8844 ± 3094	10021 ± 3602	8791 ± 3442	0.0742
**Neutrophils count, x10** ^**3**^ **cells/ml**	6877 ± 2672	6189 ± 1895	7541 ± 3190	6741 ± 3097	0.060
**Lymphocytes count, x 10** ^**3**^ **cells/ml**	1695 ± 1694	1968 ± 2291	1653 ± 1255	1364 ± 970	0.152
**Sodium, mEq/l**	138 ± 4	137 ± 5	138 ± 5	137 ± 5	0.233
**Potasium, mEq/l**	4.3 ± 0.4	4.2 ± 0.6	4.3 ± 0.3	4.4 ± 0.5	0.075
**NT-proBNP, pg/ml** ^**a**^	2803 (1384–5265)	3523 (2213–7595)	4050 (2323–6519)	6153 (2728–17855)	<0.001
**CA125, U/ml** ^**a**^	22.5 (14–36.5)	159 (111–218)	31 (19–49)	128.5 (92–180)	<0.001
**Creatinine at admission, mg/dL**	1.0 ± 0.3	0.9 ± 0.3	1.3 ± 0.6	1.4 ± 0.6	<0.001
**Urea, mg/dL**	51.7 ± 16.8	47.3 ± 25.2	62.7 ± 27.1	67.9 ± 36.1	<0.001
**eGFR, mL/min/1.73 m** ^**2**^	72 ± 20	80 ± 27	69 ± 24	60 ± 27	<0.001
**Galectin-3, ng/mL** ^**a**^	15.8 (13.8–16.6)	15.2 (13.1–16.5)	27.7 (21.3–34.8)	26.8 (21.9–37.8)	<0.001
**CRP, mg/L**	25.5 ± 35.7	18.2 ± 17.9	29.9 ± 37.4	28.8 ± 40.6	0.334
**Echocardiography**					
**LVEF, %**	56 ± 14	46 ± 17	50 ± 15	47 ± 16	0.036
**LAD, mm**	40 ± 7	43 ± 4	43 ± 7	44 ± 8	0.246
**TAPSE, mm**	20 ± 3	18 ± 4	19 ± 4	18 ± 4	0.015
**Previous treatment**					
**Previous Diuretic treatment, n (%)**	20 (62.5)	23 (53.5)	73 (72.3)	60 (68.2)	0.171
**Previous ACEI treatment, n (%)**	9 (28.1)	6 (13.9)	27 (26.7)	17 (19.3)	0.253
**Previous ARB treatment, n (%)**	11 (34.4)	15 (34.9)	28 (27.7)	22 (25.0)	0.591
**Previous Beta-blockers treatment, n (%)**	16 (50)	15 (34.9)	41 (40.6)	39 (44.3)	0.569
**Treatment** ^b^					
**Beta-blockers, n (%)**	25 (78.1)	38 (88.4)	69 (68.3)	62 (70.4)	0.058
**ACEI/ARB, n (%)**	24 (75)	32 (74.4)	61 (60.4)	63 (71.6)	0.191
**Aldosterone receptor blocker, n (%)**	12 (37.5)	24 (55.8)	28 (27.7)	35 (39.8)	0.015
**Spironolactone, n (%)**	8 (25.0)	18 (41.9)	21 (20.8)	29 (32.9)	0.054
**Eplerenone, n (%)**	5 (15.6)	6 (13.9)	7 (6.9)	6 (6.8)	0.299
**Inotropes, n (%)**	0	0	3 (3)	1 (1.1)	0.692

Galectin-3/CA125 categories: C1 = Galectin-3 ≤17.8 ng/ml & CA125 ≤67 U/ml; C2 = Galectin-3 ≤17.8 ng/ml & CA125 >67 U/ml; C3 = Galectin-3 >17.8 ng/ml & CA125 ≤67 U/ml, and C4 = Galectin-3 >17.8 ng/ml & CA125 >67 U/ml.

ACEI: angiotensin converting enzyme inhibitors; AHF: acute heart failure; ARB: angiotensin II receptor blockers; CA125: antigen carbohydrate 125; COPD: chronic pulmonary obstructive disease; CRP: C-reactive protein; DBP: diastolic blood pressure; eGFR: estimated glomerular filtration rate; ICD: implantable cardioverter defibrillator; LAD: left atrial diameter; LOS: length of stay; LVEF: left ventricular ejection fraction; NT-proBNP: amino-terminal pro-brain natriuretic peptide; PAD: peripheral artery disease; SBP: systolic blood pressure; TAPSE: tricuspid annular plane systolic excursion; and, WHO: World Health Organization.

Values for continuous variables are expressed as mean ± standard deviation.

^a^Value expressed as the median (percentile 25—percentile 75)

^b^Administered at discharge or during hospitalization in case of in-hospital deaths.

### Association among Gal-3, inflammatory status and CA125

We found that Gal-3 did not correlate with cytokines, white blood cells, C-reactive protein or red blood cells within those patients with CA125 values equal/below median (Table 2). Conversely, Gal-3 was associated with greater pro-inflammatory status in patients with CA125 above median. Indeed, Gal-3 significantly correlated with increased levels of IL-6, TNF-α, Hs-CRP and relative lymphocyte count (Table 2).

**Table 2:** Correlations between Logarithm of Galectin-3 and inflammatory biomarkers according CA125.

	CA125 ≤67 U/ml		CA125 >67 U/ml	
	r	p	r	p
**Log IL-6**	0.053	0.541	0.325	<0.001
**Log TNF-α**	-0.077	0.380	0.292	0.001
**Log IL-1b**	0.006	0.453	0.142	0.105
**Log IL-10**	0.119	0.174	0.149	0.090
**Log Hs-CRP**	0.147	0.091	0.196	0.025
**Log Relative lymphocyte count**	-0.055	0.533	-0.199	0.024

CA125: antigen carbohydrate 125; Log: logarithm; IL-6: interleukin-6; TNF-α: tumor necrosis factor α; IL-1β: interleukin-1β; IL-10: interleukin-10; Hs-CRP: High sensitivity-C-reactive protein.

### Association between the levels of Gal-3, CA125 and adverse outcomes

#### Mortality

**Fig 1:**
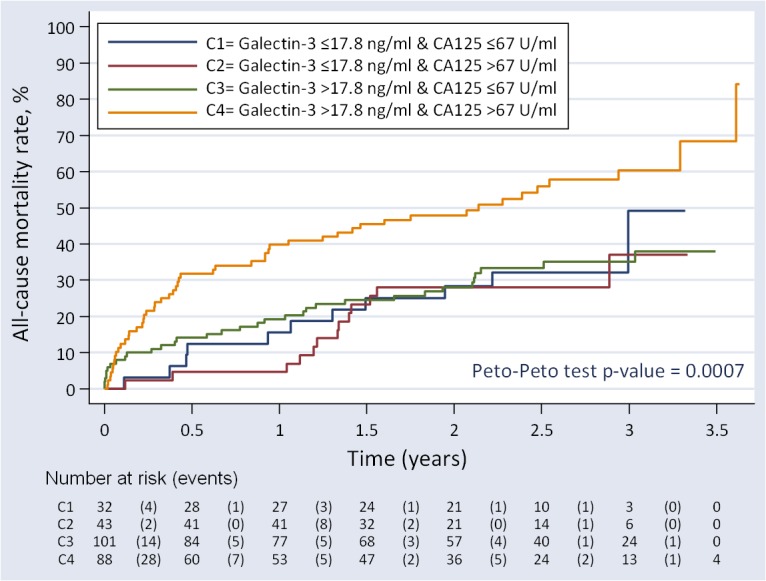
Kaplan-Meier curve depicting the cumulative mortality rates across CA125 and galectin-3 categories. CA125: antigen carbohydrate 125.

**Fig 2:**
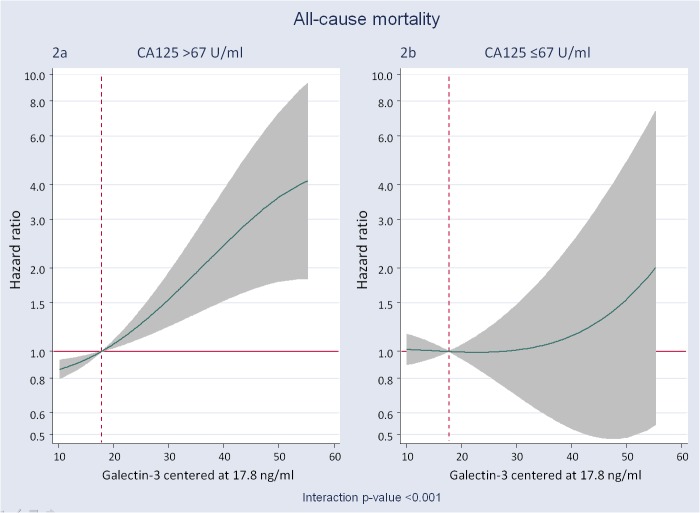
Galectin-3 and its relation with the risk of all-cause mortality expressed as adjusted hazard ratios. Gal-3 was modelled linearly with the gradient of risk, and centred at a threshold of risk of 17.8 ng/ml. A) Gal-3 and the risk of all-cause mortality in patients with CA125 >67 U/ml; B) Gal-3 and the risk of all-cause mortality in patients with CA125 ≤67 U/ml. CA125: antigen carbohydrate 125; Gal-3: galectin-3.

During a median follow-up of 2 years (1–2.8) 108 (40.9%) deaths were recorded. Levels of Gal-3 and CA125 were significantly higher in those patients who died as compared with those who remained alive [median (IQR): 26.1 (18.8–36.5) vs. 20.8 (16.6–28.1), p<0.001 and 86.5 U/ml (49–158) vs. 51.2 U/ml (21.4–119), p<0.001, respectively]. The crude mortality rates were estimated in 1.66, 1.49, 1.67 and 3.67 x 10 person-years for C1, C2, C3 and C4, respectively (p<0.001). Kaplan-Meier curve revealed higher risk for patients with both biomarkers elevated (C4) with significant separation in the curves during the first 6 months of follow-up (Fig 1). In multivariable setting, Gal-3 and CA125_median were independently associated to higher mortality (P_Gal-3_ = 0.006 and P_CA125_ = 0.004). Gradient of risk along the continuum of Gal-3 and CA125 are shown in S1A and S1B Fig, respectively. Further analysis revealed that the risk of mortality attributable to Gal-3 was modified according to CA125_median values (p-value for interaction<0.001). In the presence of CA125 >67 U/ml, the association between Gal-3 and mortality is best described as a positive and almost linear relationship (Fig 2A). Such trajectory translated into HRs ranging from 1.06 (95% CI: 1.02–1.10) to 3.60 (95% CI: 1.67–7.72) for values of Gal-3 between 20 and 50 pg/ml and using a reference threshold at 17.8 pg/ml. In contrast, Gal-3 lacked any significant effect on mortality in the group of patients with CA125 levels ≤67 U/ml (Fig 2B). In this same multivariate scenario, by combining the information of CA125 (dichotomized at the median) and Gal-3 [dichotomized at 17.8 ng/ml or at the median (22.3 ng/ml)], patients with both biomarkers elevated showed an independent increase of risk of mortality compared to those with high Gal-3 but CA125 below the median (Table 3).

**Table 3:** CA125/Gal-3 categories and risk of adverse events.

**All-cause mortality**			
	**Adjusted HR (CI 95%), p-value**		**Adjusted HR (CI 95%), p-value**
**Gal-3 >17.8 ng/ml and CA125 ≤67 U/ml**	1	Gal-3 >22.3 ng/ml and CA125 ≤67 U/ml	1
**Gal-3 >17.8 ng/ml and CA125 >67 U/ml**	2.16 (1.29–3.64) p = 0.004	Gal-3 ≤22.3 ng/ml and CA125 >67 U/ml	1.91 (1.06–3.43) p = 0.032
**Gal-3 ≤17.8 ng/ml and CA125 ≤67 U/ml**	1.30 (0.66–2.53) p = 0.445	Gal-3 >22.3 ng/ml and CA125 ≤67 U/ml	0.79 (0.41–1.51) p = 0.478
**Gal-3 ≤17.8 ng/ml and CA125 >67 U/ml**	1.59 (0.84–3.01) p = 0.151	Gal-3 >22.3 ng/ml and CA125 >67 U/ml	1.46 (0.74–2.88) p = 0.272
**Repeated hospitalizations**			
	**Adjusted IRR (CI 95%), p-value**		**Adjusted IRR (CI 95%), p-value**
**Gal-3 >17.8 ng/ml and CA125 ≤67 U/ml**	1	Gal-3 >22.3 ng/ml and CA125 ≤67 U/ml	1
**Gal-3 >17.8 ng/ml and CA125 >67 U/ml**	1.53 (1.07–2.19) p = 0.019	Gal-3 ≤22.3 ng/ml and CA125 >67 U/ml	1.42 (0.96–2.10) p = 0.077
**Gal-3 ≤17.8 ng/ml and CA125 ≤67 U/ml**	1.06 (0.64–1.76) p = 0.822	Gal-3 >22.3 ng/ml and CA125 ≤67 U/ml	0.96 (0.62–1.46) p = 0.838
**Gal-3 ≤17.8 ng/ml and CA125 >67 U/ml**	0.87 (0.57–1.34) p = 0.525	Gal-3 >22.3 ng/ml and CA125 >67 U/ml	1.07 (0.69–1.66) p = 0.768

CA125: carbohydrate antigen 125; Gal-3: galectin-3; HR; hazard ratio; IRR: incident rate ratio.

Similar differential prognostic effect of Gal-3 across CA125 levels were found when the analysis was restricted to CV-mortality.

#### Hospital readmissions

**Fig 3:**
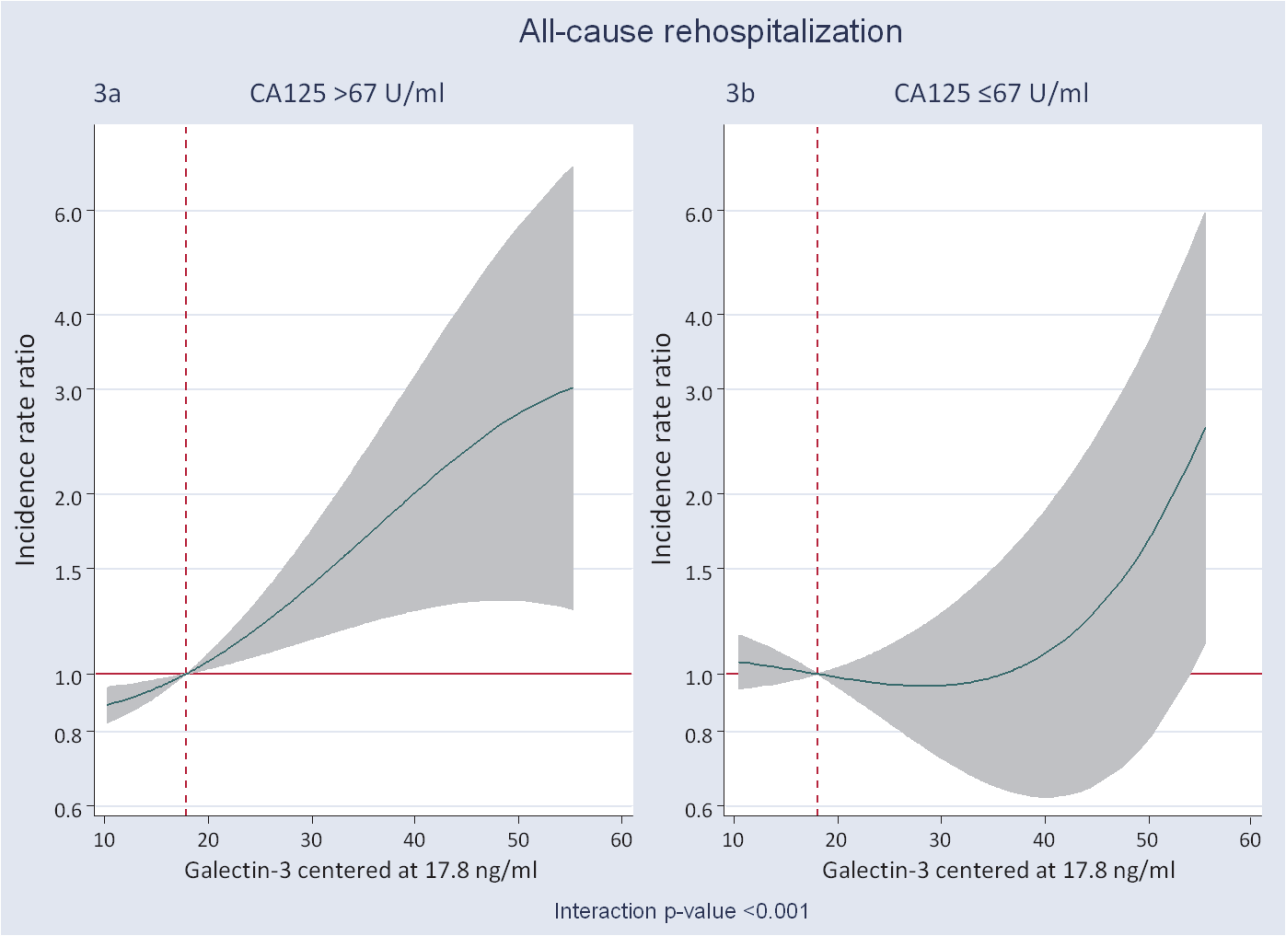
Galectin-3 and its relation with the risk of all-cause rehospitalization expressed as incidence rate ratios. Gal-3 was modelled linearly with the gradient of risk, and centered at a threshold of risk of 17.8 ng/ml. A) Gal-3 and the risk of all-cause rehospitalization in patients with CA125 >67 U/ml. B) Gal-3 and the risk of all-cause rehospitalization in patients with CA125 ≤67 U/ml. CA125: antigen carbohydrate 125; Gal-3: galectin-3. This differential prognostic effect was also observed when CV readmissions were evaluated as endpoint (Fig 4B).

For the analysis of re-hospitalization, only patients discharged alive were considered (n = 246). At a median follow-up of 2.05 years (IQR: 1.23–2.86), 365 all-cause rehospitalizations in 171 patients (69.5%) were recorded and distributed as follow: 1 = 77 (29.2%); 2 = 40 (15.1%); 3 = 27 (10.2%); 4 = 14 (5.3%); 5 = 9 (3.4%); 6 = 3 (1.1%) and 8 = 1 (0.4%). Most of re-hospitalizations were due to cardiovascular conditions (58.9%) including AHF as the most frequent condition (43.8%). The crude re-hospitalization rates for C1, C2, C3 and C4 categories were estimated in 0.70, 0.51, 0.79 and 0.89 per person-years, respectively. In the whole sample and tested individually, Gal-3 was positively associated to higher risk of readmission (P_gal-3_ = 0.009) as is shown in S2A Fig. Similarly, CA125 was positively but borderline associated to higher risk (S2B Fig). Multivariable analysis also revealed a significant interaction between Gal-3 and CA125_median (p<0.001). The direction of the effect mimicked the mortality endpoint. In fact, Gal-3 was positively associated to higher risk of repeated re-admissions (Fig 3A) only in the group of patients with CA125 >67 U/ml. Values in between 20 to 50 pg/ml were related to an excess of risk for re-admission ranging from an IRR of 1.07 (95% CI: 1.02–1.12) to 2.74 (95% CI: 1.33–5.65). On the other hand, Gal-3 did not show any relationship with the risk of re-admission in those patients with CA125 levels ≤ 67 U/ml (Fig 3B). Similarly, when Gal-3 was dichotomized at 17.8 ng/ml or at median (22.3 ng/ml) multivariate estimates revealed that, compared to patients with high Gal-3 but CA125 below median, only patients with CA125 and Gal-3 elevated were independently associated to an increased risk of repeated readmission (Table 3).

**Fig 4:**
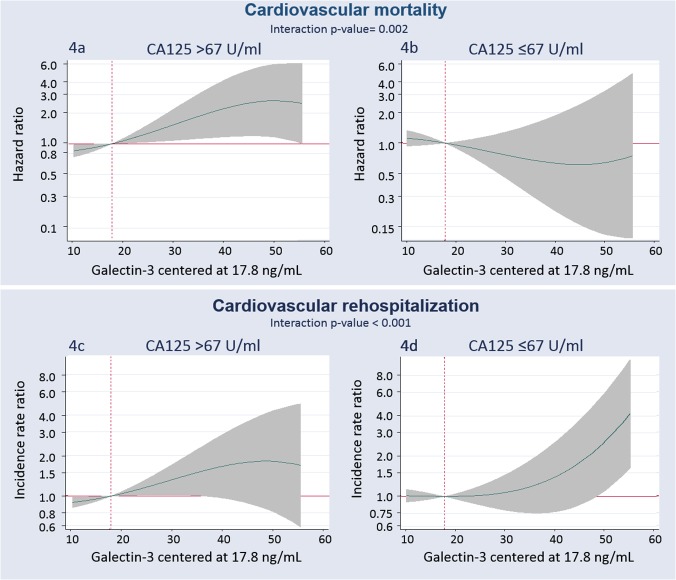
Galectin-3 and its relation with the risk of mortality and repeated readmissions due to cardiovascular causes. Gal-3 was modelled linearly with the gradient of risk, and centered at a threshold of risk of 17.8 ng/ml. A) Gal-3 and the risk of cardiovascular mortality in patients with CA125 >67 U/ml. B) Gal-3 and the risk of cardiovascular mortality in patients with CA125 ≤67 U/ml. C) Gal-3 and the risk of cardiovascular rehospitalization in patients with CA125 >67 U/ml. D) Gal-3 and the risk of cardiovascular rehospitalization in patients with CA125 ≤67 U/ml. Estimates for cardiovascular mortality adjusted for age, etiology, prior admission for acute heart failure, left ventricular ejection fraction<50%, glomerular filtration rate, NT-pro brain natriuretic peptide, treatment with beta blocker and treatment with angiotensin converting enzyme inhibitor. Estimates for cardiovascular rehospitalization adjusted for age, etiology, prior admission for acute heart failure, left ventricular ejection fraction, pleural effusion, prior stroke, prior myocardial infarction, implantable cardiac defibrillator, blood urea nitrogen, NT-pro brain natriuretic peptide, and treatment with beta blocker. Gal-3: galectin-3; CA125: antigen carbohydrate 125.

## Discussion

In the present study we demonstrate for the first time that the prognostic effect of Gal-3 in patients with AHF was differentially determined by CA125. Indeed, the prognostic effect attributable to Gal-3 was greater in those patients exhibiting higher values of CA125 (above median: >67 U/ml), while it did not reach significance in those patients with CA125 concentrations ≤67 U/ml. This unexpected pattern of association suggests a possible mechanistic link between CA125 and Gal-3 not only as a biomarkers in AHF but also as functional binding partners during AHF-related inflammatory response. Whether this association is derived from a truly biological interaction between these two molecules, or merely reflects some degree of overlapping in pathophysiologic pathways still remains to be elucidated.

### Galectin-3 and CA125 as risk markers

Gal-3 is a biomarker expressed by several tissues [1, 8–10], including myocardium, where it has been shown to profoundly influence cardiac remodelling [1], probably by acting on several pathophysiologic processes, such as inflammation and collagen turn-over [1]. Indeed, several experimental studies have identified important roles for Gal-3, an endogenous lectin, not only as a bystander, but as a critical mediator of cardiac remodelling [1, 6, 16]. In fact, the deleterious effects of Gal-3 on cardiac remodelling are clinically supported by findings showing that higher levels of this lectin may be independently related to the severity of HF, and to higher risk of adverse outcomes in both, acute and chronic settings [1–3, 14].

Likewise, plasma CA125 has been reported to be elevated in most of patients with AHF, and positively correlated to systemic congestion, inflammation, and severity of the disease [4, 5, 7, 17]. In addition, higher levels of this glycoprotein have been consistently associated to worse outcomes independently of traditional risk factors such as natriuretic peptides and cardio-renal indexes [4, 5, 18]. Interestingly, a number of studies have suggested the clinical utility of both biomarkers for monitoring and guiding therapy in AHF setting [3, 5, 19–21].

### Pathophysiology

Gal-3 is an endogenous chimera-type lectin bearing a carbohydrate-recongnition binding domain that enables binding and cross-linking of β-galactosides-containing glycoconjugates (8–10). The deleterious effects of Gal-3 are, at least in part, attributed to the capacity of this protein to bind matrix proteins forming a “mesh-like” shaped configuration, which contributes to increase the mass and stiffness of the intercellular matrix [1]. Moreover, Gal-3 has proposed to be a key pro-inflammatory factor that contributes to Th1 and Th17-polarized immune responses and Gal-3-deficient (*Lgals3* ^*-/-*^) mice display reduced sensitivity to autoimmune and chronic inflammatory responses [22, 23]. These findings place Gal-3 as a key regulator of inflammatory responses, although their precise roles in cardiac inflammation and the mechanisms underlying these effects have not yet been elucidated. In this regard, Gal-3 has been shown to be essential for early wound healing and ventricular remodelling after myocardial infarction in mice, suggesting a major role for this lectin in cardiac physiology [24].

Although the biological relevance of Gal-3-CA125 interactions is still uncertain, we might speculate that these two critical biomarkers could functionally associate to regulate inflammatory responses and tissue remodelling through glycosylation-dependent mechanisms. Interestingly, CA125 is a giant mucin-like glycoprotein, that through poorly understood mechanisms, a) facilitates progression and metastasis of ovarian cancer, b) acts as a barrier for trophoblast adherence to the endometrium, c) protects cancer cells of NK cell-mediated destruction; d) controls erythrocyte aggregation and; e) serves as a barrier for bacterial and viral infections in ocular epithelium [12, 25, 26]. Nevertheless, the precise biological role of this glycoprotein in cardiac biology still remains uncertain. Experimental evidence revealed that CA125-associated *N*-glycans are implicated in recognition events involved in both innate and adaptive arms of immune responses [12] Along with this line, CA125 has been demonstrated to serve as a lectin counter receptor showing higher affinity for Gal-1 and Gal-3 [10]. In this regard, structural analysis of O-glycans decorating CA125 revealed the presence of both core type 1 and type 2 glycans, which, upon extension of lactosamine moieties, constitute relevant ligands for Gal-3 binding and function. On the other hand, CA125 is also *N*-glycosylated, expressing complex bisecting type *N*-linked glycans, which are also key ligands that facilitate Gal-3 recognition [27]. Importantly, previous findings indicated that the cellular milieu in which CA125 is expressed may also have a significant impact by conferring selectivity to galectin binding [10]. Illustrating this concept, the effects of CA125 on erythrocytes and corneal cells have been reported to be differentially controlled by interactions with Gal-1 or Gal-3 irrespectively [25, 26].

In HF, increased levels of CA125 are frequently found during acute decompensation, asa result of multifactorial mechanisms [17]. The available evidence suggests that CA125 is synthesized by serosal cells in response to mechanical stress and/or inflammatory stimuli [28]. *In vitro* experiments have shown that inflammatory mediators including IL-1, TNF-α and lipopolysaccharides can stimulate the secretion of CA125 from mesothelial cells [29].

Based on the current knowledge, a number of explanations could be postulated. First, in the heart, Gal-3 exerts a fibrogenic effect in contrast to the anti-fibrogenic activity of Gal-1 [1, 30]. Multivalent carbohydrates, selectively cross-linked by structurally-different Gal-3 (pentamer) and Gal-1 (dimer) could explain, at least in part, the antagonic effects of these closely-related lectins in heart pathophysiology [31, 32]. We speculate that the conformational structural changes as well as the selective glycosylation patterns of CA125 could confer greater avidity for the Gal-3 pentamer as compared with the Gal-1 dimer resulting in a predominant ‘pro-inflammatory’ effect Gal-3 over an ‘anti-inflammatory’ effect Gal-1. In fact, we found that in patients with higher values of CA125, Gal-3 significantly correlated with surrogates of inflammation; whereas this effect did not occur in those individuals with CA125 equal/below median. An alternative scenario regarding a possible functional CA125-Gal-3 association is that this interaction could occur in a multivalent fashion and generate strong molecular lattices that are highly resistant to lateral movement increasing the mass and stiffness of the intercellular matrix [33]. Further studies are warranted to explore these possibilities and dissect the physiologic relevance of Gal-3-CA125 interactions with the overarching goal of proposing Gal-3-CA125 interaction as a therapeutic target in AHF.

Of note, there are some limitations associated with this study that deserve to be mentioned: a) this is a single-center observational study which, by design, can lead to residual (and unmeasured) confounding factors; b) the possibility that the sample size could be insufficient to test the effect of these interaction with an appropriate statistical power; c) the low sample size precludes to evaluate this differential prognostic effect is observed in the most representative subgroups of the disease, and; d) although this study was not designed to explore the dynamic and interrelationship of these two biomarkers at molecular levels, it has allowed the postulation of possible hypotheses that could provide rational explanations for the interdependence of Gal-3 and CA125 in AHF.

## Conclusion

We conclude that, in patients with AHF, the prognostic effect of Gal-3 is dictated by the levels of CA125. In fact, its deleterious effect was specifically observed in the subgroup of patients belonging to the upper median of CA125. Further studies are warranted to confirm these results and to elucidate their pathophysiologic relevance.

## Supporting Information

S1 Fig Galectin-3 and CA125 and the risk of all-cause mortality (main effects). CA125: carbohydrate antigen 124.
(TIF)

S2 Fig Galectin-3 and CA125 and the risk of rehospitalizations (main effects). CA125: carbohydrate antigen 124.
(TIF)
