# Supplementary figures and images for: Prognostic Value of the Interaction between Galectin-3 and Antigen Carbohydrate 125 in Acute Heart Failure
Source: PLoS One. 2015 Apr 13;10(4):e0122360. doi: 10.1371/journal.pone.0122360 (PMC4395409; doi:10.1371/journal.pone.0122360)

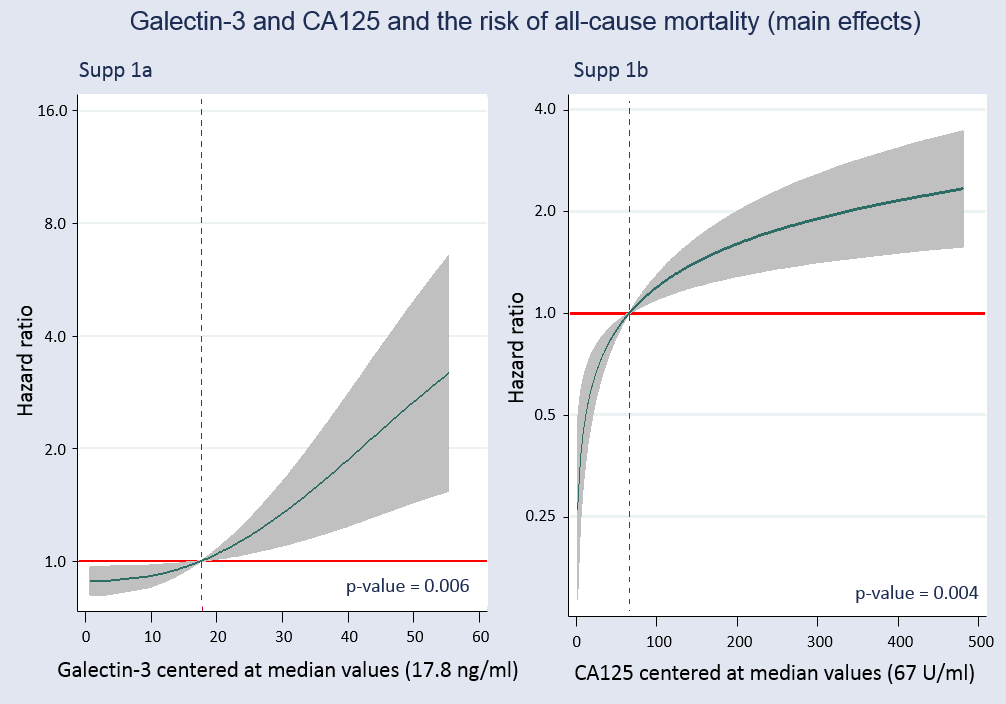

Supplement: S1 Fig — CA125: carbohydrate antigen 124. (TIF) [file pone.0122360.s001.tif]

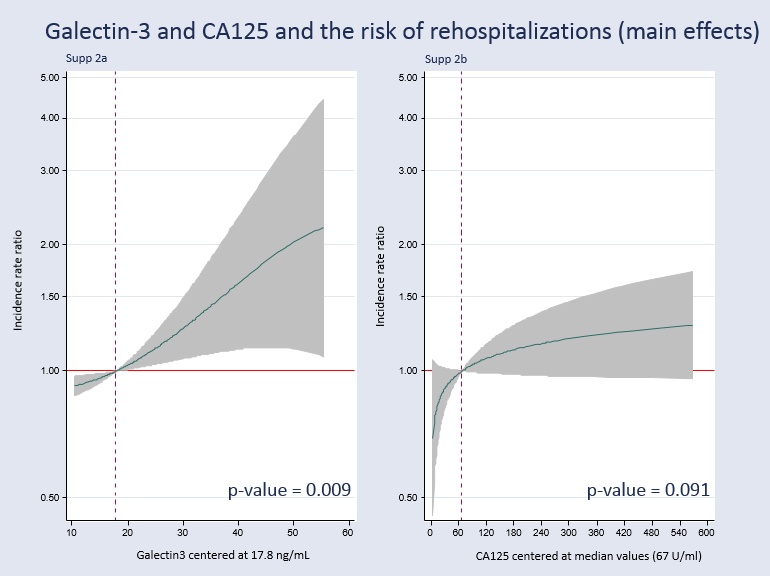

Supplement: S2 Fig — CA125: carbohydrate antigen 124. (TIF) [file pone.0122360.s002.tif]
